# The definition and detection of acute kidney injury

**DOI:** 10.12861/jrip.2014.08

**Published:** 2013-11-30

**Authors:** John W Pickering, Zoltán H Endre

**Affiliations:** ^1^Department of Medicine, University of Otago Christchurch, Christchurch, New Zealand; ^2^Department of Nephrology, Prince of Wales Clinical School, University of New South Wales, Sydney, Australia

**Keywords:** Acute kidney injury, Glomerular filtration rate, Cystatin C

## Abstract

The first consensus definition of Acute Kidney Injury (AKI) was published a decade ago. In this mini narrative review we look at the history of the changes in the definition of AKI and consider how it may change again in the near future. The epidemiology of small changes in creatinine and the difficulties with determining baseline creatinine have driven the changes. Recent evidence on urinary output and the application of structural injury biomarkers are likely to change the definition once more.

**Implication for health policy/practice/research/medical education:**

The first consensus definition of Acute Kidney Injury (AKI) was published a decade ago. In this mini narrative review we look at the history of the changes in the definition of AKI and consider how it may change again in the near future. The epidemiology of small changes in creatinine and the difficulties with determining baseline creatinine have driven the changes. Recent evidence on urinary output and the application of structural injury biomarkers are likely to change the definition once more.

## Introduction

In ancient times the Persian philosopher Avicenna noted that urine may be retained in crisis of fever (s393) and prescribed hot oil baths (s413) (1). Unfortunately, apart from the supportive therapy of dialysis, there has been little progress since in the treatment of acute kidney injury (AKI). One of the impediments to progress has been the lack of a standardised AKI definition. This in part may explain why AKI is under-recognized (2). What follows is a narrative review of the attempts to define this syndrome over the past 10 years and our speculation on where we expect definitions to change based on our own research and that of other scientists.

## Consensus to evidence

The Acute Quality Dialysis Initiative (ADQI) met in 2003 to produce the first consensus definition of acute renal failure (ARF) (3). For the sake of clarity we will use AKI, a term introduced a few years later, rather than ARF throughout this article. Known as the Risk, Injury, Failure, Loss of kidney function and End Stage Kidney Disease (RIFLE) definition this was a very important first step towards providing a platform by which comparative epidemiology and clinical outcomes could be judged. RIFLE proposed an AKI classification scheme based on one of two criteria, a glomerular filtration rate (GFR) criteria or urine output criterion (Table 1). The first three levels, R, I, or F, were levels of renal dysfunction which could be assessed either by the GFR or urine output criteria. Grades, L and E, are clinical outcomes following diagnosis of AKI.

**Table 1:** Acute kidney injury consensus definitions

**Definition**	**GFR**	**Creatinine surrogate**	**Urine Output**
RIFLE (2004)	Decrease >25%*	Increase ≥50% from baseline • Recommended to use MDRD with eGFR of 75-100 ml/min/1.73m^2^ to estimate baseline creatinine when missing. • To be determined over 7 days.	<0.5 ml/kg/h for 6 hours
AKIN (2007>)	-	Increase ≥0.3 mg/dl (26.4 umol/l) or ≥50% within 48 hours. • No baseline needed, but two measures within 48 hours needed	<0.5 ml/kg/h for more than 6 hours
KDIGO (2012)	-	Increase ≥0.3 mg/dl (26.4 umol/l) within 48 hours or ≥50% above baseline known or presumed to have occurred within the prior 7 days	<0.5 ml/kg/h for 6 hours
*Creatinine kinetics suggests this should be 33.3% to make it equivalent to a 50% increase in creatinine (4)			

The GFR criteria recognised that ARF was primarily the rapid loss of filtration function and that measurement of changes of serum creatinine was the most common means by which to assess loss of GFR. RIFLE equated a loss in GFR with an increase in serum creatinine of 50%. This was intended to be the definition of the “entry-level” ARF stage. Unfortunately, an error in creatinine kinetics was included in the definition. A 25% loss of GFR was published as purportedly equivalent to an increase in creatinine of 50%. In the Lancet in 2009 we pointed out that this is mathematically incorrect. A loss of one-third of GFR is equivalent to an increase of 50% in creatinine (4). A similar error was made by equating a 75% decrease in GFR, rather than a two-thirds decrease, to a 200% increase in creatinine for RIFLE stage F. In practice, most studies apply the serum creatinine criteria alone rather than the GFR criteria. However, some epidemiological studies and trials that have used a loss of creatinine clearance or decrease in estimated GFR to define AKI are affected by this error.

The great success of the RIFLE definition has been that each stage beginning with an increase in serum creatinine of 50% is associated with increased severity of harm, in particular mortality (5). The difficulty has been the consistency of its application. The original RIFLE publication gave no indication of the intended time period following recognised insult that serum creatinine changes of greater than 50% should be considered as AKI. An analysis of un-refereed ADQI reports and the early papers following the publication of the RIFLE criteria suggests that this was intended to be 7 days (6), which has been incorporated into the recent Kidney Disease Improving Global Outcomes (KDIGO) consensus AKI definition (7).

In 2006 the Acute Kidney Injury Network (AKIN) offered a revised definition of AKI (8). The term “acute kidney injury” was adopted in recognition that an acute decline in renal function is often secondary to injury. The AKIN definition was subtly changed from that of RIFLE by including an absolute change in creatinine of ≥ 0.3 mg/dl (≥ 26.4 umol/l) within 48 hours as part of the AKI definition. The value, 0.3 mg/dl, was partly chosen as the minimum difference that could be reliably measured by modern analysers and by its association with a 4.1 increase in odds ratio for mortality (9,10). The 50% change was retained, but duration of increase was restricted to within 48 hours. The short duration was intended to obviate the need for a baseline creatinine, but required at least two creatinine measures approximately 48 hours apart. The one-week timing of RIFLE was retained by AKIN for the severity staging of AKI. The Loss and End-Stage categories were removed from the staging of severity. Defining AKI by a change in GFR was removed and the urine output criteria remained unaltered.

The RIFLE authors acknowledged that the use of serum creatinine to define AKI depends on knowledge of an individual’s baseline, or “normal” creatinine. In many cases, there is no recent baseline creatinine measurement available. RIFLE recommended that in such situations the Modified Diet in Renal Disease (MDRD) equation be used to “back-calculate” a baseline creatinine assuming an estimated GFR (eGFR) of 75 to 100 ml/min/1.73m^2^. Although not evidence based, this strategy was rapidly adopted and is still used today. In 2009-2010 several papers emerged which assessed the evidence for this recommendation. In a cohort of AKI patients, defined by markers other than creatinine, Bagshaw and colleagues demonstrated that in chronic kidney disease (CKD) patients the use of the MDRD formula to define baseline creatinine resulted in an overestimation of the incidence of AKI (11). Our own study in a cohort of ICU patients demonstrated that MDRD to estimate baseline creatinine performed no better than randomly generating baseline creatinine along a log-normal distribution (12). We found that compared with a known baseline creatinine there was a systematic increase in error at higher baseline creatinine. Using the lowest creatinine in the ICU to estimate baseline improved the results. We also showed that the CKD-EPI equation resulted in similar errors. In other cohorts, other studies have revealed similar problems with the RIFLE suggestion (13,14). Considerable effort has been made to find a solution to this issue. Siew and colleagues have shown that the mean creatinine within a year of AKI event is best where baselines are available and that multiple-imputation methods provide more accurate estimates of baseline creatinine than MDRD back-calculation(15,16). We have taken a more pragmatic approach and suggested that for epidemiological studies or trial analysis that a post-discharge (within 90 days) or, if unavailable, lowest of discharge or on-admission creatinine be used where no suitable pre-admission creatinine is available (17). This, of course, does not help diagnosis on patient presentation.

The recent KDIGO definition is a combination of AKIN and RIFLE (7). It defined AKI by a ≥ 0.3 mg/dl increase in creatinine within 48 hours, or a ≥50% *above baseline* within 7 days. From a practical point of view this enabled diagnosis of AKI using the 0.3 mg/dl criterion even when baseline creatinine is unknown (as did AKIN), but allowed for slower developing changes in creatinine (as did RIFLE). Changes were made to the AKIN severity stage 3 to enable incorporation of the pediatric population into both the AKI definition and staging. The urine output criteria remained unaltered. KDIGO has also explicitly made clear that this definition of AKI is for all cases including pediatrics and contrast induced nephropathy, which have traditionally applied alternate definitions.

## New evidence, new directions

There has been little effort to verify the urine output criteria. Consistently, more patients are diagnosed with AKI when the urine output criterion is applied than when only the change in creatinine criterion is applied. Macedo and colleagues in a single centre study showed that urine output of <0.5 ml/kg/h for 6 hours is associated with increased mortality even in the absence of a change in creatinine (18). We have recently investigated in an intensive care unit cohort whether 0.5 ml/kg/h for 6 hours is the optimum threshold for defining AKI (19). We discovered that between 0.3 and 0.5 ml/kg/h for 6 hours there was no difference in mortality compared with urine output >0.5 ml/kg/h for 6 hours. When urine output fell below 0.3 ml/kg/h for 6 hours there was a marked increase in mortality. The same increase in mortality could be observed in urine output was even lower for shorter duration. We developed an equation to suggest that AKI could be defined when urine output (ml/kg/h) was less than [0.3 × (duration of collection (hours)) + 0.11], although it is questionable whether an output recorded for less than 2 hours has sufficient specificity.

The duration over which creatinine remains elevated is associated with severity of outcome. Brown and colleagues demonstrated that post cardiac surgery the duration of AKI is directly proportional to long-term mortality (20). Coca and colleagues demonstrated a similar relationship in a non-cardiac surgery cohort of diabetics (21). Indeed, the mortality rate of 3 to 6 days of AKI stage 1 was greater than 2 or fewer days of AKI stage 3. While even a transient change in creatinine is associated with increased injury biomarkers (22), we suggest that duration of elevation of surrogate markers of function should be incorporated into the severity staging of AKI.

Within the ICU it has been postulated that the practise of giving large boluses of fluid may dilute creatinine and thereby delay the diagnosis of AKI (23). We combined a creatinine-kinetic model with a volume-kinetic model and demonstrated that there is likely to be significant dilution very shortly following a fluid bolus, but that this alone will only delay diagnosis by a few hours (24). However, the fluid accumulation will result in an underestimation of AKI severity. More importantly, we observed that following cardiac arrest in the majority of patients the initial-post arrest creatinine concentration dropped dramatically and much more than could be explained by dilution. In patients with no change in creatinine the outcomes were worse and we observed elevated levels of kidney injury biomarkers in the urine. The data suggest that the cardiac arrest may temporarily cease the production of creatinine. On the basis of this study and the observations in animal models that sepsis inhibits creatinine production (25) and that creatinine production is reduced in dialysis patients (26), Prowle raised provocative questions about the use of creatinine to determine drug dosing, need for RRT, and in biomarker studies (27).

Plasma cystatin C is a possible alternative surrogate for changes in kidney function to creatinine. Using a rise of cystatin C or of creatinine of 50% as the threshold for AKI in a cohort of 85 patients, 44 of whom had AKI, Herget-Rosenthal demonstrated that an increase in cystatin C of 50% occurred 1.5±0.6 days earlier than creatinine (28). In a paired analysis in 444 ICU patients we found that while cystatin C was effective and earlier than creatinine at diagnosing AKI the difference was only about 6 hours (29).

A short duration creatinine clearance can also be used to help diagnose AKI. Normally this is not measured prior to an event. Therefore it must either be used in conjunction with an estimated baseline creatinine clearance using the Cockcroft-Gault formula or similar, or to indicate whether or not renal function has recovered when this is yet to be reflected in a change in serum creatinine (30).

The search for biomarkers of renal injury has been driven by a desire for markers of AKI that are elevated earlier than serum creatinine following a loss of GFR and therefore enable earlier intervention (31). Most of the analysis of injury biomarkers has been to compare them to AKI defined by a change in creatinine. This process has seen some biomarkers, such as Neutrophil Gelatinase Associated Lipocalin (NGAL) appear as candidates for the tag “early biomarker.” However, the discovery and validation process has been hampered by the comparison with creatinine, which is like an obsession of comparing apples with oranges (32). Most of these biomarkers reflect structural injury or repair and are not direct elevated merely because of changes in GFR. Any association with creatinine, is therefore because the injury reflected by the elevated injury biomarker causes a loss of GFR later reflected by increased creatinine. In a seminal paper, Haase and colleagues demonstrated that an increased NGAL is associated with increased mortality even when an increase in creatinine is not observed (33). Recently, ADQI have recommended that structural injury biomarkers be incorporated into AKI definitions (34,35). We have suggested that if a biomarker above a specific threshold is to be considered equivalent to a particular KDIGO stage then it must be similarly prognostic of meaningful clinical outcomes (36). We demonstrated that a biomarker threshold for AKI diagnosis could be determined by first calculating the sensitivity of the creatinine-based AKI definition for need for dialysis or death within 30 days. The biomarker threshold is therefore the threshold that has this same sensitivity for need for dialysis or death within 30 days. An alternative would be analogous to the use of troponin and that is to define the threshold for AKI as the 99^th^ percentile of the normal concentration (37).

## Conclusion

The diagnosis of Acute Kidney Injury has progressed significantly since the first consensus definition (RIFLE). Evidence has been provided for the definition of AKI and severity staging. This evidence has resulted in the addition of a small absolute change of creatinine over a short duration. Progress has been made about how best to estimate baseline creatinine to enable diagnosis. This, though, is not fully resolved. Future definitions, Table 2, may incorporate cystatin C as an alternative marker to creatinine, but the expense of implementing this is not yet justified by evidence of superiority. This may change if treatments are developed that require implementation of earlier diagnosis and in light of further revelations of where and when creatinine fails as a surrogate for changes in GFR. The urine output criteria may change in the face of new evidence. Indeed, Ravi Mehta has already called for changes in the application of urine output in AKI suggesting that the lower threshold we found be used for diagnosis (38). The association of severity of outcome with duration of elevation of AKI could be incorporated within severity stage definitions by allowing patients with longer durations below the current higher severity stage thresholds to be incorporated into those stages. Current efforts to develop near real-time measures of GFR (39-41) may result in GFR returning to the definition of AKI (42). Structural injury biomarkers of AKI are almost certain to be incorporated into the definition of AKI. We hope and expect that this will precipitate renewed efforts into early and effective treatments for the syndrome.

**Table 2:** A possible future AKI definition

**Functional**			**Damage**
**GFR or Creatinine clearance**	**Creatinine or Cystatin C surrogate**	**Urine Output**	
Decrease >33.3%	Creatinine increase ≥0.3 mg/dl (26.4 umol/l) within 48 hours or ≥50% above baseline known or presumed to have occurred within the prior 7 days OR Cystatin C increase ≥50% above baseline known or presumed to have occurred within the prior 7 days*	<0.3 ml/kg/h for 6 hours OR anuria for 1 hour	Biomarker >99th percentile of normal OR Biomarker >threshold for mortality or need for dialysis with same sensitivity as creatinine definition.
* It would be expected that an absolute increase in cystatin C over a short duration would be associated with increased mortality analogous to the absolute increase in creatinine. However, the threshold for this change to be associated with a similar odds ratio for mortality has yet to be determined.

## Author contributions

JWP wrote the first draft. JWP and ZHE refined the manuscript.

## Conflict of interests

The authors declared no competing interests.

## Ethical consideration

Ethical issues (including plagiarism, misconduct, data fabrication, informed consent, double publication) have been completely observed by the authors.

## Funding/Support

None.
